# Real‐world prevalence and prognostic significance of hepatotoxicity after chimeric antigen receptor T‐cell therapy

**DOI:** 10.1111/bjh.70677

**Published:** 2026-07-07

**Authors:** Julius Hollnberger, Tim Richardson, Philipp Gödel, Joshua Leutiger, Jan Christoph Schumacher, Sophie Schiestel, Benedict Allhoff, Christof Scheid, Udo Holtick, Christoph Neumann‐Haefelin, Gabriel Allo, Philipp Kasper

**Affiliations:** ^1^ Department of Gastroenterology and Hepatology, University Hospital Cologne and Faculty of Medicine University of Cologne Cologne Germany; ^2^ Department 1 of Internal Medicine, University Hospital Cologne and Faculty of Medicine University of Cologne Cologne Germany

**Keywords:** CAR T‐cell therapy, cytokine release syndrome, hepatotoxicity, liver function tests, real‐world study

## Abstract

In this real‐world cohort of 231 patients receiving chimeric antigen receptor (CAR) T‐cell therapy, hepatotoxicity occurred in 58% of patients, predominantly as transient transaminase elevations, and was not associated with impaired 12‐month overall survival (adjusted hazard ratio [HR] 1.18, 95% confidence interval [CI] 0.71–1.94).

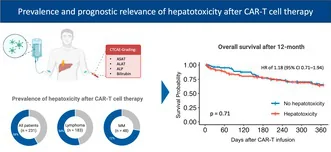

To the Editor,

Chimeric antigen receptor (CAR) T‐cell therapy has become an established treatment option for patients with relapsed or refractory haematological malignancies, particularly lymphoma and multiple myeloma, where it can induce long‐term remissions and improve survival outcomes.^1^, ^2^, ^3^, ^4^, ^5^ Frequent complications include immune effector cell‐related toxicities such as cytokine release syndrome (CRS), immune effector cell‐associated neurotoxicity syndrome (ICANS) and immune effector cell‐associated haemophagocytic lymphohistiocytosis‐like syndrome, as well as haematological and infectious complications.^6^, ^7^, ^8^ More recently, second primary malignancies and adverse cutaneous events have been recognised as additional late toxicities of CAR‐T‐cell treatment.^9^, ^10^, ^11^ However, while most side effects are relatively well known and characterised, less attention has been paid to organ‐specific toxicities beyond the nervous and haematopoietic systems. Elevations in liver function tests have been reported as part of general adverse event summaries, with incidences ranging from 14% to 77%, often in temporal association with CRS. However, existing reports are predominately descriptive and do not address prognostic implications of abnormal liver functions tests after CAR‐T‐cell treatment.^12^ We therefore aimed to systematically characterise the prevalence, timing and severity of hepatotoxicity in a real‐world CAR‐T cohort and explore its association with clinical outcomes.

This retrospective single‐centre cohort study included 231 patients with lymphoma (*n* = 183) or multiple myeloma (*n* = 48) who received CAR‐T‐cell therapy at the University Hospital of Cologne between 2018 and 2024. The analysis included patients who had at least one hepatic laboratory parameter—aspartate aminotransferase (ASAT), alanine aminotransferase (ALAT), alkaline phosphatase (ALP) or total bilirubin—available within 14 days before to 30 days after infusion. Hepatotoxicity was graded according to Common Terminology Criteria of Adverse Events (CTCAE v5.0) with sex‐adjusted upper limits of normal at baseline (day −14 to −1) and within an early (day 0–14) and late (day 15–30) window in relation to CAR‐T infusion. A pre‐lymphodepletion sensitivity window (day −14 to −6) was additionally analysed. Survival was analysed by Kaplan–Meier estimation and Cox regression. A multivariable model was performed including age, disease entity and hepatic comorbidity. Full statistical methods and sensitivity analyses are provided in the Supporting Information.

The median age of the cohort was 61.5 years and 62.3% were male. Most patients in both cohorts had progressive disease at the time of infusion. Patients with lymphoma had received fewer prior therapies than patients with multiple myeloma (median 3 vs. 7, *p* < 0.0001). Diffuse large B‐cell lymphoma was the most frequent lymphoma subtype (131/183; 71.6%) (Table S1). Documented hepatic comorbidity (steatotic liver disease, prior or active viral hepatitis B/C/E, Gilbert syndrome) was present in 17/231 (7.4%), metabolic cardiovascular risk factors (type 2 diabetes, hypertension, dyslipidaemia, obesity) in 74/231 (32.0%) and cardiac comorbidity (heart failure, coronary artery disease and atrial fibrillation) in 17/231 (7.4%) of patients. Exposure to potentially hepatotoxic co‐medication (LiverTox category A or B) was documented in 63/226 (27.9%) of patients^13^; five patients had incomplete medication records. Baseline characteristics including CAR‐T products are summarised in Table 1.

**TABLE 1:** Baseline characteristics.

Characteristic	All patients (*n* = 231)	Lymphoma (*n* = 183)	Myeloma (*n* = 48)	*p*‐Value
Demographics and clinical characteristics				
Age, median (IQR), years	61.5 (54.0–70.6)	61.1 (52.8–71.3)	63.1 (57.4–68.8)	0.3788*
Male, *n* (%)	144/231 (62.3%)	114/183 (62.3%)	30/48 (62.5%)	0.9792^†^
Hepatic comorbidity, *n* (%)	17/231 (7.4%)	13/183 (7.1%)	4/48 (8.3%)	0.7590^§^
Metabolic/cardiovascular comorbidity, *n* (%)	74/231 (32.0%)	57/183 (31.1%)	17/48 (35.4%)	0.5726^§^
Cardiac comorbidity, *n* (%)	17/231 (7.4%)	15/183 (8.2%)	2/48 (4.2%)	0.5357^§^
Hepatotoxic co‐medication (LiverTox A/B), *n* (%)^‡^	63/226 (27.9%)	53/179 (29.6%)	10/47 (21.3%)	0.2569^§^
Disease characteristics				
Prior therapy lines, median (IQR)	3.0 (2.0–5.0) (*n* = 194^‡^)	3.0 (2.0–3.0) (*n* = 146^‡^)	7.0 (5.0–9.0) (*n* = 48)	<0.0001*
Disease status at infusion, *n* (%)				<0.0001*
CR	25/231 (10.8%)	25/183 (13.7%)	0/48 (0%)	—
VGPR	8/231 (3.5%)	0/183 (0%)	8/48 (16.7%)	—
PR	18/231 (7.8%)	16/183 (8.7%)	2/48 (4.2%)	—
SD	18/231 (7.8%)	12/183 (6.6%)	6/48 (12.5%)	—
PD	124/231 (53.7%)	92/183 (50.3%)	32/48 (66.7%)	—
No available data	38/231 (16.5%)	38/183 (20.8%)	0/48 (0.0%)	—
CAR‐T product, *n* (%)				
Tisa‐cel	75/231 (32.4%)	75/183 (41.0%)	—	
Axi‐cel	41/231 (17.8%)	41/183 (22.4%)	—	
Liso‐cel	29/231 (12.6%)	29/183 (15.8%)	—	
Brexu‐cel	9/231 (3.9%)	9/183 (4.9%)	—	
Investigational^¶^	29/231 (12.6%)	29/183 (15.8%)	—	
Ide‐cel	24/231 (10.4%)	—	24/48 (50.0%)	
Cilta‐cel	24/231 (10.4%)	—	24/48 (50.0%)	
Baseline parameters at infusion				
CRP, median (IQR), mg/dL	6.3 (2.1–27.8) (*n* = 202^‡^)	6.7 (2.0–35.4) (*n* = 157^‡^)	5.9 (2.3–13.6) (*n* = 45^‡^)	0.4255*

*Note*: Data are presented as median (interquartile range) or *n* (%). *p*‐values compare lymphoma versus myeloma using *Mann–Whitney *U*‐test, ^†^chi‐squared or ^§^Fisher exact test, as appropriate. ^‡^Values are reported for patients with available data; variable‐specific denominators are provided in the table. No imputation was performed for missing clinical baseline variables. Comorbidity data were available for *n* = 131 patients; for the remaining patients, comorbidities were coded as absent (conservative assumption). Hepatic comorbidity includes steatosis hepatis/non‐alcoholic fatty liver disease, hepatitis B, hepatitis E and Gilbert syndrome. Metabolic/cardiovascular comorbidity includes diabetes mellitus, arterial hypertension, hyperlipidaemia and obesity. Cardiac comorbidity includes heart failure, coronary artery disease and atrial fibrillation. Hepatotoxic co‐medication defined as LiverTox category A or B; *n* = 5 patients with missing co‐medication data excluded. ^¶^Investigational products include anti‐CD19 (*n* = 9), anti‐CD20 (*n* = 9) and combined anti‐CD19/CD20 (*n* = 1) constructs.

Abbreviations: Axi‐cel, Axicabtagene ciloleucel (CD19); Brexu‐cel, Brexucabtagene autoleucel (CD19); CAR‐T, chimeric antigen receptor T‐cell therapy; Cilta‐cel, Ciltacabtagene autoleucel (BCMA); CR, complete response; CRP, C‐reactive protein; Ide‐cel, Idecabtagene vicleucel (BCMA); IQR, interquartile range; Liso‐cel, Lisocabtagene maraleucel (CD19); *n*, number of patients; PD, progressive disease; PR, partial response; SD, stable disease; Tisa‐cel, Tisagenlecleucel (CD19); VGPR, very good partial response.

Abnormal liver function tests (LFTs) were detected in 39.1% of patients at baseline. Following CAR‐T‐cell therapy, the prevalence increased to 58.0% during the early post‐infusion phase (day 0–14; *p* < 0.0001 vs. baseline) and decreased to 42.0% during the late phase (day 15–30; *p* = 0.8590 vs. baseline) (Figure S1). Median severity was CTCAE grade 1, while severe hepatotoxicity (grade ≥3) occurred in 8.0% of patients. Two patients with lymphoma experienced hepatotoxicity grade 4, both of whom died within 10 days after CAR‐T‐cell infusion due to ICANS with concurrent CRS grade 4 and CRS complicated by sepsis with abdominal abscesses respectively. None of the fatal cases were attributable to isolated liver failure.

Abnormal LFTs were more frequent in myeloma than in lymphoma patients (72.2% vs. 54.2%; odds ratio [OR] 2.20, 95% confidence interval [CI] 1.05–4.87; *p* = 0.0306) (Table S2). Rates of LFT abnormalities did not differ significantly across LiverTox A/B exposure (*p* = 0.6252), hepatic comorbidity (*p* = 0.8972) or metabolic cardiovascular risk factors (*p* = 0.8360) (Table S3). A lower rate of LFT abnormalities was observed in patients with cardiac comorbidity (*p* = 0.0078, *n* = 17).

After accounting for pre‐existing baseline abnormalities, 33.0% of patients experienced a worsening in CTCAE grade during the early phase (day −14 to −1), predominantly involving transaminases (ALAT 28.1%; ASAT 29.1%) rather than ALP (12.2%) or bilirubin (8.6%) (Table S4). Lymphodepleting chemotherapy was initiated per in‐house protocol on day −5 before CAR‐T‐cell infusion. A sensitivity analysis using a pre‐lymphodepletion baseline window (day −14 to −6) yielded a lower proportion of patients with abnormal baseline LFTs (23.5% vs. 38.9%). This consequently led to a higher proportion of patients showing a worsening of LFT during the early phase (41.5% vs. 33.0%) (Table S5).

Early‐phase hepatotoxicity (CTCAE ≥1) was not associated with deteriorated 12‐month overall survival. In a multivariable Cox model adjusted for age, disease entity and baseline hepatic comorbidity (226 patients, 64 events), the adjusted hazard ratio (HR) was 1.18 (95% CI 0.71–1.94; *p* = 0.5287) (Figure 1). The unadjusted HR was 1.10 (95% CI 0.67–1.81; *p* = 0.7047; log‐rank *p* = 0.7056). Entity‐stratified analyses were concordant (lymphoma log‐rank *p* = 0.5779; myeloma log‐rank *p* = 0.7411); a formal interaction test did not indicate effect‐measure modification by entity (*p* for interaction = 0.8992). An uncensored sensitivity analysis including all events beyond 365 days (78 events) yielded consistent results (HR 0.90, 95% CI 0.57–1.41; *p* = 0.6481). Survival analyses of patients with worsening LFTs were concordant irrespective of the baseline window definition (primary: HR 0.96, 95% CI 0.56–1.62, *p* = 0.8652; sensitivity: HR 0.99, 95% CI 0.60–1.64, *p* = 0.9838) (Figure S2). In a post hoc exploratory restricted mean survival time (RMST) analysis, RMST was shorter within the first 120 days among patients with early hepatotoxicity (adjusted RMST difference −8.10 days; 95% CI −15.70 to −0.51; *p* = 0.0365), with no difference at the prespecified 365‐day horizon (−13.55 days; 95% CI −48.51 to 21.41; *p* = 0.4478).

**FIGURE 1:**
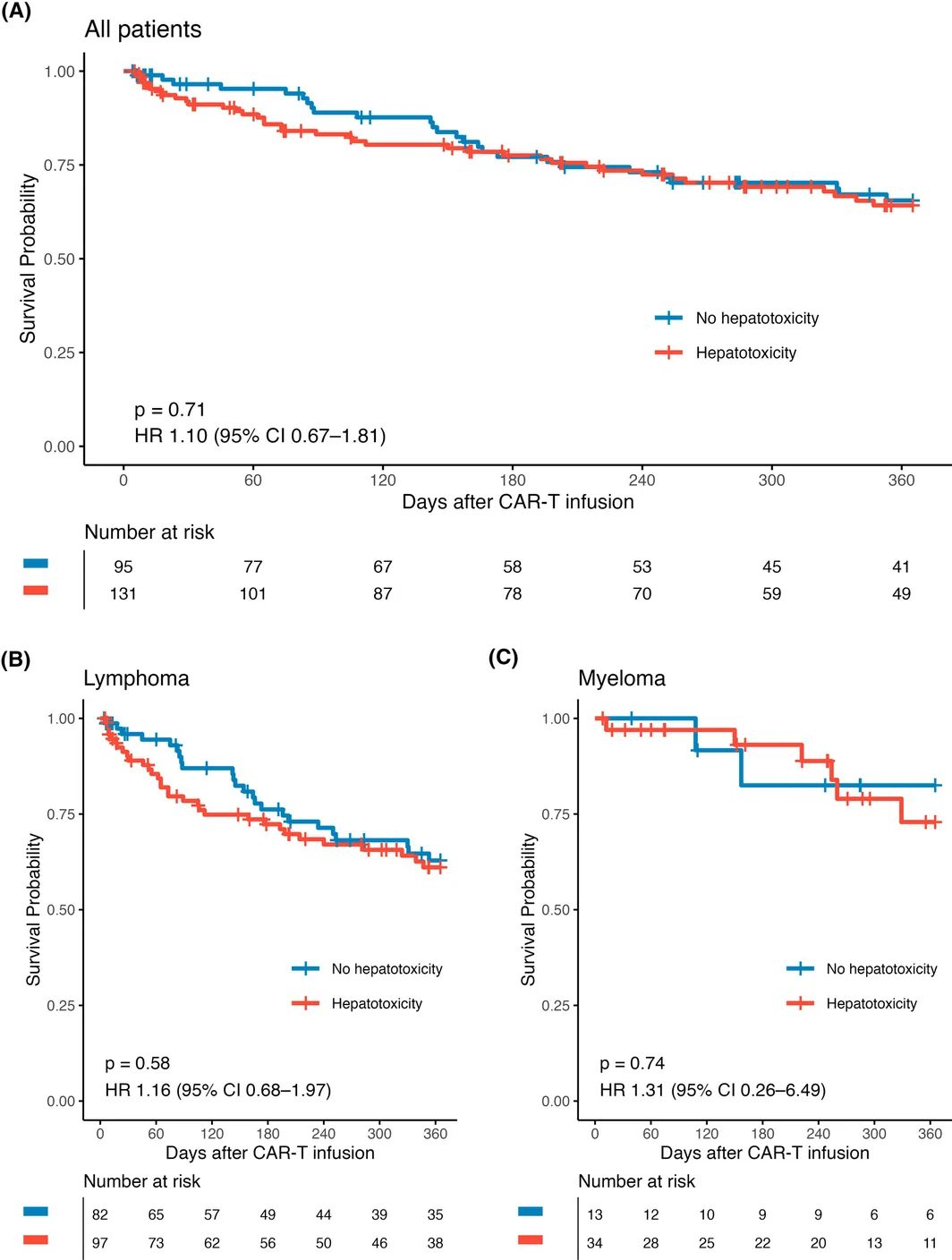
Early‐phase hepatotoxicity after CAR‐T‐cell infusion was not associated with reduced survival at 365 days. Kaplan–Meier survival curves comparing overall survival between patients with and without hepatotoxicity in the early phase after CAR‐T‐cell infusion (days 0–14) where survival data were available. Hepatotoxicity was defined as a maximum composite CTCAE grade ≥1 across ALAT, ASAT, ALP and bilirubin per CTCAE v5.0. Analysis shown for (A) all patients, (B) lymphoma cohort and (C) myeloma cohort (*n* = 226, 179 and 47, with 64, 56 and 8 events, respectively, during 365‐day follow‐up). Hazard ratios with 95% confidence intervals are displayed within each panel. *p*‐values were calculated using the log‐rank test. Risk tables show the number of patients at risk at specified time intervals. ALAT, alanine aminotransferase; ALP, alkaline phosphatase; ASAT, aspartate aminotransferase; CAR‐T, chimeric antigen receptor T cell; CI, confidence interval; CTCAE, Common Terminology Criteria for Adverse Events; HR, hazard ratio; *n*, number of patients.

CRS occurred in 67.5% of patients. Detailed timing was available for the lymphoma cohort, with a median time to CRS onset of 2 days (interquartile range [IQR] 1–5) and a median duration of 4 days (IQR 2–7) post‐infusion. Hepatotoxicity was more frequent in patients with CRS than without (64.2% vs. 43.1%; OR 2.37, 95% CI 1.29–4.39; *p* = 0.0037). CRS grade correlated weakly with hepatotoxicity severity (Spearman's *ρ* = 0.22, 95% CI 0.09–0.34; *p* = 0.0009), confined to transaminase elevations in lymphoma patients (Table S6).

This real‐world cohort demonstrates that LFT abnormalities were common (58.0%), occurring predominantly early and transiently following infusion, but were not associated with an impaired 12‐month overall survival. Multivariable adjustment for age, disease entity and hepatic comorbidity yielded consistent results.

The observed prevalence of LFT abnormalities exceeded that reported in pivotal registration trials (16%–31%), likely reflecting broader inclusion criteria, less selected patient populations and more granular laboratory monitoring in real‐world practice.^1^, ^2^, ^3^, ^4^, ^5^ Abnormal LFTs were more frequent in myeloma than lymphoma patients, consistent with prior reports and likely reflecting the higher cumulative treatment burden and different CAR‐T constructs used in this population.^1^, ^2^, ^3^, ^4^, ^5^ The heterogeneity of CAR‐T products did not allow specific analysis and warrants further research.

Exposure to potentially hepatotoxic co‐medications (LiverTox category A or B) and relevant comorbidity domains did not identify any factor significantly associated LFT abnormalities. Interestingly, a significantly lower rate was observed in patients with cardiac comorbidity (*p* = 0.0078), though this subgroup comprised only 17 patients; further studies are required. Taken together, these findings did not identify pre‐existing comorbidity‐related or co‐medication‐driven factors as major contributors to LFT abnormality prevalence, though moderate confounding effects cannot be excluded given the study's sample size.

In our cohort, hepatotoxicity overlapped temporally with CRS, but the correlation between CRS grade and hepatotoxicity severity was weak and confined to lymphoma patients, supporting temporal co‐occurrence rather than a consistent causal relationship. Mechanistically, CAR‐T‐associated hepatotoxicity likely arises from a convergence of factors: (i) systemic cytokine‐mediated injury during CRS, (ii) direct hepatotoxicity from lymphodepleting chemotherapy, in particular cyclophosphamide, for which transient transaminase elevations have been described, and (iii) potentially off‐tumour on‐target effects of CAR‐T constructs reported rarely in registry safety data.^13,^, ^14^, ^15^ Exploratory post hoc RMST analyses suggest a time‐limited early mortality signal within the first 120 days that warrants prospective evaluation but should be interpreted cautiously as the number of events was small and the formal interaction test did not support entity‐specific effect modification (*p* = 0.8992).

Strengths of this study include the comparatively large real‐world cohort, the high temporal resolution of LFT data allowing differentiation of early and late post‐infusion dynamics, as well as the systematic evaluation of hepatotoxic co‐medications and comorbidity domains as confounding factors, which has not been previously reported in this context, to our knowledge. Limitations include the retrospective single‐centre design, variable data availability across parameters and residual confounding and the absence of uniformly available biomarkers of systemic inflammation or laboratory data beyond 30 days. The potential impact of bridging therapy could not be evaluated due to insufficient data availability.

In summary, hepatotoxicity after CAR‐T‐cell therapy is common, occurs predominantly early after infusion and was not associated with impaired overall survival at 12 months in this real‐world cohort. Exploratory analyses suggest an association with CRS and a possible time‐limited early vulnerability, underscoring the need for prospective studies to further clarify the clinical relevance of early liver function abnormalities.

## AUTHOR CONTRIBUTIONS

**Julius Hollnberger:** Conceptualization; methodology; validation; visualization; formal analysis; writing – original draft; writing – review and editing; data curation; investigation. **Tim Richardson:** Data curation; writing – review and editing. **Philipp Gödel:** Data curation; writing – review and editing. **Joshua Leutiger:** Writing – review and editing; methodology; investigation; validation. **Jan Christoph Schumacher:** Investigation; validation; methodology; writing – review and editing. **Sophie Schiestel:** Methodology; validation; investigation; writing – review and editing. **Benedict Allhoff:** Methodology; investigation; writing – review and editing; validation. **Christof Scheid:** Writing – review and editing; project administration. **Udo Holtick:** Project administration; writing – review and editing. **Christoph Neumann‐Haefelin:** Writing – review and editing; project administration; supervision; resources. **Gabriel Allo:** Methodology; conceptualization; validation; writing – review and editing; investigation; writing – original draft. **Philipp Kasper:** Conceptualization; methodology; writing – review and editing; writing – original draft; validation; investigation.

## FUNDING INFORMATION

The authors declare that no funding was received for the conduct of this study.

## CONFLICT OF INTEREST STATEMENT

JH, PG, JL, JCS, SS, BA, CN‐H, GA and PK have nothing to disclose. TR has received honoraria (not related to this study) for advisory board participation and/or speaker engagements from Takeda, Johnson & Johnson, Sanofi, Jazz Pharmaceuticals and Bristol Myers Squibb (BMS). Research funding was provided by Oncopeptides (not related to this study). CS gave advisory boards and received honoraria (not related to this study) from Amgen, Abbvie, Bristol‐Myers Squibb, Janssen, Novartis, Oncopeptides, Pfizer, Roche, Sanofi, Stemline Menarini and Takeda, and received research support (not related to this study) from Janssen and Takeda. UH has received honoraria (unrelated to this study) for BMS, Kite/Gilead, Johnson&Johnson, Miltenyi Biotec, Novartis.

## ETHICS STATEMENT

The study was conducted in accordance with the Declaration of Helsinki and approved by the local Ethics Committee of the Faculty of Medicine, University of Cologne (reference: 25‐1130‐retro).

## PATIENT CONSENT STATEMENT

All patients provided informed consent to the use of their anonymised data at the time of admission.

## Supporting information


**Figure S1.** Hepatic CTCAE grade distribution before and after CAR‐T‐cell infusion.
**Figure S2.** Worsening of hepatotoxicity after CAR‐T infusion is not associated with impaired overall survival.
**Table S1.** Frequency of diagnosis of lymphoma patients.
**Table S2.** (a–c) Frequency of CTCAE grade by patient cohorts, time window and laboratory parameters.
**Table S3.** Association of potential confounders with hepatotoxicity severity in the early post‐infusion phase.
**Table S4.** Proportion of patients with worsening CTCAE grade in the early phase (0–14 days) after CAR‐T therapy compared to baseline.
**Table S5.** Sensitivity analysis: impact of baseline window definition on the association between hepatotoxicity worsening and overall survival.
**Table S6.** Correlation between CRS grade and severity of hepatotoxicity in the early phase after CAR‐T‐cell infusion.

## Data Availability

No individual‐level patient data will be made available for sharing.
