# Supplementary material for: Real‐world prevalence and prognostic significance of hepatotoxicity after chimeric antigen receptor T‐cell therapy
Source: Br J Haematol. 2026 Jul 7;209(3):1404–9. doi: 10.1111/bjh.70677 (PMC13570130; doi:10.1111/bjh.70677)
Supplement: Supplementary file 1 [file BJH-209-1404-s001.docx]

**Supporting information**

## Methods:

**Patients and data collection:**

This retrospective, single-center study included consecutive patients who received CAR T-cell therapy at the University Hospital of Cologne between 2018 and 2024. Patients were eligible for inclusion if regular laboratory assessments were available and at least one hepatic laboratory parameter of interest – aspartate aminotransferase (ASAT), alanine aminotransferase (ALAT), alkaline phosphatase (ALP), or total bilirubin – was measured within the interval from 14 days before to 30 days after CAR-T cell infusion. Longitudinal analyses comparing baseline and post-infusion liver function were performed in patients with available baseline measurements and at least one follow-up assessment. Patients were followed for up to 12 months after infusion. All patients were screened for chronic hepatitis B and C; all lymphoma patients additionally underwent baseline PET imaging without hepatic involvement. Demographic, clinical, laboratory, and outcome data were extracted from the electronic medical records. Patients were excluded if (i) no laboratory data were available within the predefined time window (−14 to +30 days) or (ii) they had previously received CAR-T cell therapy.

The study was conducted in accordance with the Declaration of Helsinki and was approved by the Ethics Committee of the Faculty of Medicine, University of Cologne (reference: 25-1130-retro).

**Definitions and classification of laboratory parameters:**

Hepatotoxicity was graded according to the National Cancer Institute Common Terminology Criteria for Adverse Events (CTCAE), version 5.0 (1), based on abnormalities in ASAT, ALAT, ALP, and total bilirubin. Laboratory-specific, sex-adjusted upper limits of normal (ULN) were applied: 35 U/L for ASAT and ALAT in females and 50 U/L in males; 104 U/L for ALP in females and 129 U/L in males; and 1.2 mg/dL for total bilirubin irrespective of sex.

Three time windows were defined: baseline (14 days before infusion to the day of CAR-T cell administration), early (day 0–14), and late (day 15–30). For each parameter and time window, the maximum CTCAE grade was determined by identifying the highest grade among all available measurements. A composite liver toxicity grade, referred to as overall maximum CTCAE grade, was defined as the highest CTCAE grade observed across the four hepatic laboratory parameters within a given time window.

Lymphodepleting chemotherapy was initiated per institutional standard operating procedure on day −5 before CAR-T cell infusion. To account for potential LFT changes attributable to lymphodepleting chemotherapy within the primary baseline window (day −14 to −1), a sensitivity analysis using a pre-lymphodepletion baseline window (day −14 to −6) was performed in parallel.

The primary outcome was the prevalence of hepatotoxicity, defined as CTCAE grade ≥1. Analyses assessing associations between hepatotoxicity, cytokine release syndrome, and survival outcomes were considered exploratory. CRS was graded according to established consensus criteria and managed in accordance with institutional standard operating procedures aligned with current American Society of Clinical Oncology guidelines.

**Confounder and comorbidity definitions**

Hepatic comorbidity was defined as the presence of any of the following prior to CAR-T infusion: hepatic steatosis, prior or active hepatitis B or C, prior hepatitis E, or Gilbert syndrome. Metabolic cardiovascular risk factors were defined as the presence of type 2 diabetes, arterial hypertension, dyslipidaemia, or obesity. . Cardiac comorbidity was defined as the presence of at least one of the following: heart failure (HFrEF/HFpEF), coronary artery disease, or atrial fibrillation/flutter.

For each patient, all co-medications documented at the time of CAR-T cell infusion were individually assessed against the LiverTox database and assigned a hepatotoxicity likelihood category (A through E). Each patient was then assigned the highest (most hepatotoxic) category across all their co-medications. Patients classified as category A (well-documented hepatotoxicity) or B (probable hepatotoxicity) based on this maximum category assignment were considered to have exposure to potentially hepatotoxic co-medication. Exposure was defined as documented administration of any LiverTox category A or B agent within the study time window. Prophylactic agents administered to all patients per institutional standard operating procedure — specifically cotrimoxazole (trimethoprim-sulfamethoxazole) and valaciclovir — were excluded from the co-medication analysis, as their uniform administration across the cohort precludes differential confounding. Five patients had incomplete medication records and were excluded from this analysis.

**Statistical Analysis:**

Continuous variables are reported as medians with interquartile ranges (IQR), and categorical variables as frequencies and percentages. Baseline characteristics between lymphoma and myeloma patients were compared using the Mann–Whitney U test for continuous variables and the chi-square test or Fisher exact test for categorical variables, as appropriate. Ordinal variables (e.g. disease status at infusion) were compared using the Wilcoxon rank-sum test.

Changes in CTCAE grades across time windows were analysed using the Stuart–Maxwell test for marginal homogeneity. Paired LFT comparisons were performed using the Wilcoxon signed-rank test; to account for multiple testing, false discovery rate correction was applied using the Benjamini–Hochberg method.

Overall survival was analysed using Kaplan–Meier estimates with log-rank tests for group comparisons. A multivariable Cox proportional hazards model was pre-specified including age, disease entity and hepatic comorbidity as covariates, with a formal interaction test for effect-measure modification by entity (hepatotoxicity × entity interaction term). An uncensored sensitivity analysis was performed including all events beyond 365 days (78 events vs. 64 in the primary analysis) to assess the influence of administrative censoring on survival estimates. Restricted mean survival time (RMST) analyses were performed for 120-day and 365-day horizons and adjusted for age, sex and disease entity; these were considered exploratory. Spearman rank correlation coefficients were used to assess associations between laboratory parameters and CRS severity; correlation analyses were restricted to subgroups of at least 10 patients.

Statistical significance was defined as a two-sided p-value <0.05. All p-values are reported to four decimal places or as <0.0001. All analyses were performed using R version 4.3.0 and RStudio version 2023.12.1+402.

# Supplementary Tables

## Table S1. Frequency of diagnosis of lymphoma patients

| Diagnosis | Patients, n (%)  n=183 |
| --- | --- |
| DLBCL | 131 (71.6) |
| PMBCL | 7 (3.8) |
| MCL | 10 (5.5) |
| FL | 13 (7.1) |
| PCNSL | 3 (1.6) |
| NA | 19 (10.4) |
|  |  |

Abbreviations: DLBCL, diffuse large B-cell lymphoma; PMBCL, primary mediastinal B-cell lymphoma; MCL, mantle cell lymphoma; FL, follicular lymphoma; PCNSL, primary central nervous system lymphoma; NA, no entity diagnosis available

## Table S2 a-c. Frequency of CTCAE grade by patient cohorts, time window and laboratory parameters

**a. All Patients**

| Parameter | CTCAE Grade | Baseline | Early | Late | p-value |
| --- | --- | --- | --- | --- | --- |
|  |  | **n=225** | **n=226** | **n=69** |  |
| ALAT | Grade 0 | 183 (80.9%) | 137 (60.6%) | 54 (79.4%) | <0.0001 |
|  | Grade 1 | 38 (16.9%) | 66 (29.2%) | 11 (16.2%) |  |
|  | Grade 2 | 2 (0.9%) | 9 (4%) | 2 (2.9%) |  |
|  | Grade 3 | 3 (1.3%) | 13 (5.8%) | 1 (1.5%) |  |
|  | Grade 4 | 0 (0%) | 1 (0.4%) | 0 (0%) |  |
|  |  | **n=221** | **n=210** | **n=63** |  |
| ASAT | Grade 0 | 185 (83.3%) | 133 (63.3%) | 53 (84.1%) | <0.0001 |
|  | Grade 1 | 34 (15.3%) | 56 (26.7%) | 7 (11.1%) |  |
|  | Grade 2 | 3 (1.4%) | 11 (5.2%) | 2 (3.2%) |  |
|  | Grade 3 | 0 (0%) | 8 (3.8%) | 1 (1.6%) |  |
|  | Grade 4 | 0 (0%) | 2 (1%) | 0 (0%) |  |
|  |  | **n=211** | **n=201** | **n=56** |  |
| Bilirubin | Grade 0 | 202 (95.7%) | 179 (89.1%) | 52 (85.2%) | 0.0096 |
|  | Grade 1 | 5 (2.4%) | 16 (8%) | 5 (8.2%) |  |
|  | Grade 2 | 4 (1.9%) | 4 (2%) | 3 (4.9%) |  |
|  | Grade 3 | 0 (0%) | 2 (1%) | 1 (1.6%) |  |
|  | Grade 4 | 0 (0%) | 0 (0%) | 0 (0%) |  |
|  |  | **n=158** | **n=192** | **n=61** |  |
| ALP | Grade 0 | 116 (73.4%) | 136 (70.8%) | 46 (75.4%) | 0.5025 |
|  | Grade 1 | 39 (24.7%) | 49 (25.5%) | 13 (21.3%) |  |
|  | Grade 2 | 2 (1.3%) | 5 (2.6%) | 1 (1.6%) |  |
|  | Grade 3 | 1 (0.6%) | 2 (1%) | 1 (1.6%) |  |
|  | Grade 4 | 0 (0%) | 0 (0%) | 0 (0%) |  |
|  |  | **n=225** | **n=226** | **n=69** |  |
| Overall maximum | Grade 0 | 137 (60.9%) | 95 (42%) | 40 (58%) | <0.0001 |
|  | Grade 1 | 76 (33.8%) | 96 (42.5%) | 20 (29%) |  |
|  | Grade 2 | 8 (3.6%) | 17 (7.5%) | 6 (8.7%) |  |
|  | Grade 3 | 4 (1.8%) | 16 (7.1%) | 3 (4.3%) |  |
|  | Grade 4 | 0 (0%) | 2 (0.9%) | 0 (0%) |  |

**b. Lymphoma**

| Parameter | CTCAE Grade | Baseline | Early | Late | p-value |
| --- | --- | --- | --- | --- | --- |
|  |  | **n=182** | **n=179** | **n=63** |  |
| ALAT | Grade 0 | 146 (80.2%) | 112 (62.6%) | 51 (81%) | <0.0001 |
|  | Grade 1 | 31 (17%) | 50 (27.9%) | 9 (14.3%) |  |
|  | Grade 2 | 2 (1.1%) | 7 (3.9%) | 2 (3.2%) |  |
|  | Grade 3 | 3 (1.6%) | 9 (5%) | 1 (1.6%) |  |
|  | Grade 4 | 0 (0%) | 1 (0.6%) | 0 (0%) |  |
|  |  | **n=179** | **n=165** | **n=59** |  |
| ASAT | Grade 0 | 147 (82.1%) | 110 (66.7%) | 49 (83.1%) | 0.0003 |
|  | Grade 1 | 29 (16.2%) | 40 (24.2%) | 7 (11.9%) |  |
|  | Grade 2 | 3 (1.7%) | 8 (4.8%) | 2 (3.4%) |  |
|  | Grade 3 | 0 (0%) | 5 (3%) | 1 (1.7%) |  |
|  | Grade 4 | 0 (0%) | 2 (1.2%) | 0 (0%) |  |
|  |  | **n=173** | **n=157** | **n=107** |  |
| Bilirubin | Grade 0 | 165 (95.4%) | 139 (88.5%) | 48 (84.2%) | 0.0248 |
|  | Grade 1 | 5 (2.9%) | 14 (8.9%) | 5 (8.8%) |  |
|  | Grade 2 | 3 (1.7%) | 2 (1.3%) | 3 (5.3%) |  |
|  | Grade 3 | 0 (0%) | 2 (1.3%) | 1 (1.8%) |  |
|  | Grade 4 | 0 (0%) | 0 (0%) | 0 (0%) |  |
|  |  | **n=127** | **n=152** | **n=57** |  |
| ALP | Grade 0 | 96 (75.6%) | 107 (70.4%) | 43 (75.4%) | 0.3758 |
|  | Grade 1 | 28 (22%) | 38 (25%) | 12 (21.1%) |  |
|  | Grade 2 | 2 (1.6%) | 5 (3.3%) | 1 (1.8%) |  |
|  | Grade 3 | 1 (0.8%) | 2 (1.3%) | 1 (1.8%) |  |
|  | Grade 4 | 0 (0%) | 0 (0%) | 0 (0%) |  |
|  |  | **n=182** | **n=179** | **n=64** |  |
| Overall maximum | Grade 0 | 112 (61.5%) | 82 (45.8%) | 37 (57.8%) | <0.0001 |
|  | Grade 1 | 59 (32.4%) | 71 (39.7%) | 18 (28.1%) |  |
|  | Grade 2 | 7 (3.8%) | 13 (7.3%) | 6 (9.4%) |  |
|  | Grade 3 | 4 (2.2%) | 11 (6.1%) | 3 (4.7%) |  |
|  | Grade 4 | 0 (0%) | 2 (1.1%) | 0 (0%) |  |

**c. Myeloma**

| Parameter | CTCAE Grade | Baseline | Early | Late | p-value |
| --- | --- | --- | --- | --- | --- |
|  |  | **n=43** | **n=47** | **n=5** |  |
| ALAT | Grade 0 | 36 (83.7%) | 25 (53.2%) | 3 (60%) | 0.0046 |
|  | Grade 1 | 7 (16.3%) | 16 (34%) | 2 (40%) |  |
|  | Grade 2 | 0 (0%) | 2 (4.3%) | 0 (0%) |  |
|  | Grade 3 | 0 (0%) | 4 (8.5%) | 0 (0%) |  |
|  | Grade 4 | 0 (0%) | 0 (0%) | 0 (0%) |  |
|  |  | **n=43** | **n=45** | **n=4** |  |
| ASAT | Grade 0 | 38 (88.4%) | 23 (51.1%) | 4 (100%) | 0.0015 |
|  | Grade 1 | 5 (11.6%) | 16 (35.6%) | 0 (0%) |  |
|  | Grade 2 | 0 (0%) | 3 (6.7%) | 0 (0%) |  |
|  | Grade 3 | 0 (0%) | 3 (6.7%) | 0 (0%) |  |
|  | Grade 4 | 0 (0%) | 0 (0%) | 0 (0%) |  |
|  |  | **n=38** | **n=44** | **n=4** |  |
| Bilirubin | Grade 0 | 37 (97.4%) | 40 (90.9%) | 4 (100%) | 0.2231 |
|  | Grade 1 | 0 (0%) | 2 (4.5%) | 0 (0%) |  |
|  | Grade 2 | 1 (2.6%) | 2 (4.5%) | 0 (0%) |  |
|  | Grade 3 | 0 (0%) | 0 (0%) | 0 (0%) |  |
|  | Grade 4 | 0 (0%) | 0 (0%) | 0 (0%) |  |
|  |  | **n=31** | **n=40** | **n=4** |  |
| ALP | Grade 0 | 20 (64.5%) | 29 (72.5%) | 3 (75%) | 1.0000 |
|  | Grade 1 | 11 (35.5%) | 11 (27.5%) | 1 (25%) |  |
|  | Grade 2 | 0 (0%) | 0 (0%) | 0 (0%) |  |
|  | Grade 3 | 0 (0%) | 0 (0%) | 0 (0%) |  |
|  | Grade 4 | 0 (0%) | 0 (0%) | 0 (0%) |  |
|  |  | **n=43** | **n=47** | **n=5** |  |
| Overall maximum | Grade 0 | 25 (58.1%) | 13 (27.7%) | 3 (60%) | 0.0080 |
|  | Grade 1 | 17 (39.5%) | 25 (53.2%) | 2 (40%) |  |
|  | Grade 2 | 1 (2.3%) | 4 (8.5%) | 0 (0%) |  |
|  | Grade 3 | 0 (0%) | 5 (10.6%) | 0 (0%) |  |
|  | Grade 4 | 0 (0%) | 0 (0%) | 0 (0%) |  |

Time windows: baseline (-14 to -1 days), early (0-14 days) and late (15-30 days) relative to CAR-T cell infusion. CTCAE grades 0-4 based on multiples of upper limit of normal (ULN) according to CTCAE v5.0. Overall maximum represents maximum grade across all liver parameters per patient per time window. P-values from Stuart-Maxwell test (Baseline vs. Early). Maximum laboratory values used when multiple measurements available per time window. Variable-specific denominators are provided in the table.

ALAT, alanine aminotransferase; ASAT, aspartate aminotransferase; ALP, alkaline phosphatase; CAR-T, chimeric antigen receptor T-cell; CTCAE, Common terminology criteria for adverse events; n, number of patients.

## Table S3. Association of potential confounders with hepatotoxicity severity in the early post-infusion phase.

| Potential Confounder | CTCAE Grade, n (%) | | | | | p-value |
| --- | --- | --- | --- | --- | --- | --- |
|  | **Grade 0** | **Grade 1** | **Grade 2** | **Grade 3** | **Grade 4** |  |
| Hepatotoxic  co-medication |  |  |  |  |  |  |
| No (n=158) | 69 (43.7%) | 65 (41.1%) | 12 (7.6%) | 12 (7.6%) | 0 (0.0%) | 0.6252 |
| Yes (n=63) | 25 (39.7%) | 28 (44.4%) | 5 (7.9%) | 4 (6.3%) | 1 (1.6%) |  |
| Hepatic comorbidity |  |  |  |  |  |  |
| No (n=210) | 88 (41.9%) | 90 (42.9%) | 17 (8.1%) | 13 (6.2%) | 2 (1.0%) | 0.8972 |
| Yes (n=16) | 7 (43.8%) | 6 (37.5%) | 0 (0.0%) | 3 (18.8%) | 0 (0.0%) |  |
| Metabolic comorbidity |  |  |  |  |  |  |
| No (n=155) | 65 (41.9%) | 65 (41.9%) | 12 (7.7%) | 11 (7.1%) | 2 (1.3%) | 0.8360 |
| Yes (n=71) | 30 (42.3%) | 31 (43.7%) | 5 (7.0%) | 5 (7.0%) | 0 (0.0%) |  |
| Cardiac comorbidity |  |  |  |  |  |  |
| No (n=209) | 83 (39.7%) | 91 (43.5%) | 17 (8.1%) | 16 (7.7%) | 2 (1.0%) | 0.0078 |
| Yes (n=17) | 12 (70.6%) | 5 (29.4%) | 0 (0.0%) | 0 (0.0%) | 0 (0.0%) |  |
|  | | | | | | |

Overall maximum CTCAE hepatotoxicity grade during the early post-infusion phase (days 0–14) stratified by pre-specified potential confounders. The composite CTCAE grade represents the maximum grade across ALAT, ASAT, ALP, and bilirubin per patient. Hepatotoxic co-medication was defined as any LiverTox category A or B agent documented in the patient's co-medication list (2); five patients with missing co-medication documentation were excluded from this subgroup analysis (n=221). Hepatic comorbidity was defined as the presence of at least one of the following: hepatic steatosis (NAFLD/MASLD), prior or active hepatitis B, hepatitis C, prior hepatitis E, or Gilbert syndrome. Metabolic comorbidity was defined as the presence of at least one of the following: diabetes mellitus type 2, arterial hypertension, hyperlipidaemia, or obesity. Cardiac comorbidity was defined as the presence of at least one of the following: heart failure (HFrEF/HFpEF), coronary artery disease, or atrial fibrillation/flutter. Patients without documented comorbidities (n=131) were conservatively coded as absence of comorbidity. P-values were calculated using the Wilcoxon rank-sum test.

ALP, alkaline phosphatase; ALAT, alanine aminotransferase; ASAT, aspartate aminotransferase; CAR-T, chimeric antigen receptor T-cell; CTCAE, Common Terminology Criteria for Adverse Events; HFpEF, heart failure with preserved ejection fraction; HFrEF, heart failure with reduced ejection fraction; NAFLD, non-alcoholic fatty liver disease; MASLD, metabolic dysfunction-associated steatotic liver disease; n, number of patients.

Table S4. Proportion of Patients with Worsening CTCAE Grade in the Early Phase (0-14 days) After CAR-T Therapy compared to Baseline

| Parameter | | | All patients | Lymphoma | | Myeloma |
| --- | --- | --- | --- | --- | --- | --- |
| Patients with available ALAT data | | | 221 | 179 | | 42 |
| ALAT worsening, n (%) | | | 62 (28.1%) | 46 (25.7%) | | 16 (38.1%) |
| Patients with available ASAT data | | | 203 | 163 | | 40 |
| ASAT worsening, n (%) | | | 59 (29.1%) | 43 (26.4%) | | 16 (40.0%) |
| Patients with available Bilirubin data | | | 187 | 153 | | 35 |
| Bilirubin worsening, n (%) | | | 16 (8.6%) | 13 (8.6%) | | 3 (8.6%) |
| Patients with available ALP data | | | 148 | 118 | | 30 |
| ALP worsening, n (%) | | | 18 (12.2%) | 15 (12.7%) | | 3 (10.0%) |
| Patients with available composite liver parameters | | | 221 | 179 | | 42 |
| Any liver parameter worsening, n (%) | | | 73 (33.0%) | 54 (30.2%) | | 19 (45.2%) |
|  |  |  | | |  | |

**Data are shown as n (%). Worsening was defined as an increase in CTCAE grade compared with baseline during the first 14 days after CAR-T infusion. Analyses were restricted to patients with available baseline values and at least one corresponding laboratory assessment during the early post-infusion period; therefore, denominators vary across parameters and disease groups. Composite liver parameter worsening was defined as the maximum CTCAE grade observed among ALAT, ASAT, bilirubin, and ALP**

## Table S5. Sensitivity analysis: impact of baseline window definition on the association between hepatotoxicity worsening and overall survival.

|  | Primary Analysis | Sensitivity Analysis |
| --- | --- | --- |
| ****Baseline window**** | Day -14 to -1 | Day -14 to -6 |
| Rationale | Pre-specified primary window | Excludes lymphodepletion period |
| N included | 221 | 217 |
| ****Abnormal baseline LFT, n (%)**** | 86 (38.9%) | 51 (23.5%) |
| Patients with worsening, n (%) | 73 (33.0%) | 90 (41.5%) |
| Events (deaths) | 64 | 64 |
| HR (95% CI) | 0.96 (0.56–1.62) | 0.99 (0.60–1.64) |
| p-value | 0.8652 | 0.9838 |

Worsening was defined as any increase in composite CTCAE grade (ALAT, ASAT, ALP, bilirubin) from baseline to the early post-infusion window (days 0–14). The primary analysis used a baseline window of days −14 to −1; the sensitivity analysis used days −14 to −6 to exclude potential confounding by lymphodepleting chemotherapy. The higher proportion of worsening in the sensitivity analysis (41.5% vs. 33.0%) reflects the earlier and therefore lower baseline CTCAE grades when the lymphodepletion period is excluded. P-values from the log-rank test.

ALAT, alanine aminotransferase; ASAT, aspartate aminotransferase; ALP, alkaline phosphatase; CAR-T, chimeric antigen receptor T-cell; CI, confidence interval; CTCAE, Common Terminology Criteria for Adverse Events; HR, hazard ratio; LFT, liver function test; n, number of patients.

## Table S6. Correlation between CRS grade and severity of hepatotoxicity in the early phase after CAR-T cell infusion.

| Parameter | | n | Spearman ρ (95% CI) | P-value |
| --- | --- | --- | --- | --- |
| All patients | |  |  |  |
|  | Overall Maximum | 223 | 0.22 (0.09 to 0.34) | 0.0009 |
|  | ALAT | 223 | 0.17 (0.04 to 0.30) | 0.0089 |
|  | ASAT | 207 | 0.18 (0.05 to 0.31) | 0.0079 |
|  | ALP | 190 | 0.01 (-0.14 to 0.15) | 0.9189 |
|  | Bilirubin | 199 | 0.13 (-0.01 to 0.26) | 0.0751 |
| Lymphoma | |  |  |  |
|  | Overall Maximum | 179 | 0.19 (0.05 to 0.33) | 0.0097 |
|  | ALAT | 179 | 0.20 (0.06 to 0.34) | 0.0071 |
|  | ASAT | 165 | 0.17 (0.01 to 0.31) | 0.0322 |
|  | ALP | 152 | 0.03 (-0.13 to 0.19) | 0.7128 |
|  | Bilirubin | 157 | 0.14 (-0.02 to 0.29) | 0.0770 |
| Myeloma | |  |  |  |
|  | Overall Maximum | 44 | 0.14 (-0.17 to 0.42) | 0.3740 |
|  | ALAT | 44 | -0.11 (-0.39 to 0.20) | 0.4967 |
|  | ASAT | 42 | 0.11 (-0.20 to 0.40) | 0.4856 |
|  | ALP | 38 | 0.03 (-0.30 to 0.34) | 0.8773 |
|  | Bilirubin | 42 | 0.29 (-0.01 to 0.55) | 0.0610 |
|  | |  |  |  |

Correlation between CRS grades and severity of hepatotoxicity in the early phase after CAR-T cell infusion. Spearman correlations between CRS grades (graded after ASCO guidelines(3)) and severity of hepatotoxicity (overall maximum hepatic CTCAE grades) in the early phase after CAR-T cell infusion (days 0-14) for the overall cohort and by disease entity. Overall maximum includes the maximum value of the CTCAE grade of ALAT, ASAT, ALP and bilirubin within the time window. Only subgroups with ≥10 patients included. Values represent Spearman's ρ with 95% confidence intervals; P<0.05 considered significant. ALAT, alanine aminotransferase; ASAT, aspartate aminotransferase; ALP, alkaline phosphatase; CI, confidence interval; CRS, cytokine release syndrome

# Supplementary Figures

## Figure S1. Hepatic CTCAE grade distribution before and after CAR-T cell infusion. Prevalence of maximum CTCAE hepatotoxicity grades across three time-windows: Baseline (-14 to -1 days), Early (0-14 days), and Late (15-30 days) relative to CAR-T infusion. Maximum grade represents the worst CTCAE grade across all hepatic parameters (ALAT, ASAT, ALP, and bilirubin) per patient per time window. Analysis shown for (A) all patients, (B) lymphoma cohort, and (C) myeloma cohort (n = 231, 183, and 48, respectively). Colors indicate CTCAE grades 0-4.

ALP, alkaline phosphatase; ALAT, alanine aminotransferase; ASAT, aspartate aminotransferase; CAR-T, chimeric antigen receptor T-cell; CTCAE, Common Terminology Criteria for Adverse Events; n, number of patients.

Note: Y-axes are not scaled uniformly across panels and reflect the absolute patient numbers within each cohort.

## Figure S2. ****Worsening of hepatotoxicity after CAR-T infusion is not associated with impaired overall survival.**** Kaplan-Meier curves comparing 365-day overall survival between patients with and without worsening of hepatotoxicity from baseline to the early post-infusion period. Worsening was defined as any increase in composite CTCAE grade (ALAT, ASAT, ALP, or bilirubin) from the baseline window (days -14 to -1) to the early post-infusion window (days 0-14). Analysis shown for (A) all patients, (B) lymphoma cohort, and (C) myeloma cohort (n = 221, 179, and 42, with 64, 56, and 8 events, respectively). Five myeloma patients were excluded due to absence of laboratory values within the baseline window. Hazard ratios with 95% confidence intervals are displayed within each panel. P-values were calculated using the log-rank test. Risk tables show the number of patients at risk at specified time intervals.

ALP, alkaline phosphatase; ALAT, alanine aminotransferase; ASAT, aspartate aminotransferase; CAR-T, chimeric antigen receptor T-cell; CI, confidence interval; CTCAE, Common Terminology Criteria for Adverse Events; HR, hazard ratio; n, number of patients.

**References**

1.	U.S. department of health and human services. Common Terminology Criteria for Adverse Events (CTCAE) [Internet]. 2017 Nov 27. doi:https://dctd.cancer.gov/research/ctep-trials/for-sites/adverse-events/ctcae-v5-8x11.pdf

2.	LiverTox: Clinical and Research Information on Drug-Induced Liver Injury [Internet]. Bethesda (MD): National Institute of Diabetes and Digestive and Kidney Diseases; 2012 [cited 2026 May 3]. Available from: http://www.ncbi.nlm.nih.gov/books/NBK547852/ PubMed PMID: 31643176.

3.	Santomasso BD, Nastoupil LJ, Adkins S, Lacchetti C, Schneider BJ, Anadkat M, et al. Management of Immune-Related Adverse Events in Patients Treated With Chimeric Antigen Receptor T-Cell Therapy: ASCO Guideline. J Clin Oncol. 2021 Dec 10;39(35):3978–92. doi:10.1200/JCO.21.01992
